# Supplementary material for: Structure of Type-I Mycobacterium tuberculosis fatty acid synthase at 3.3 Å resolution
Source: Nat Commun. 2018 Sep 24;9:3886. doi: 10.1038/s41467-018-06440-6 (PMC6155276; doi:10.1038/s41467-018-06440-6)
Supplement: Supplementary file 1 — Supplementary Information [file 41467_2018_6440_MOESM1_ESM.pdf]

Supplementary Information

Structure of Type-I *Mycobacterium tuberculosis* Fatty Acid Synthase at 3.3 Å Resolution

N.Elad et al.

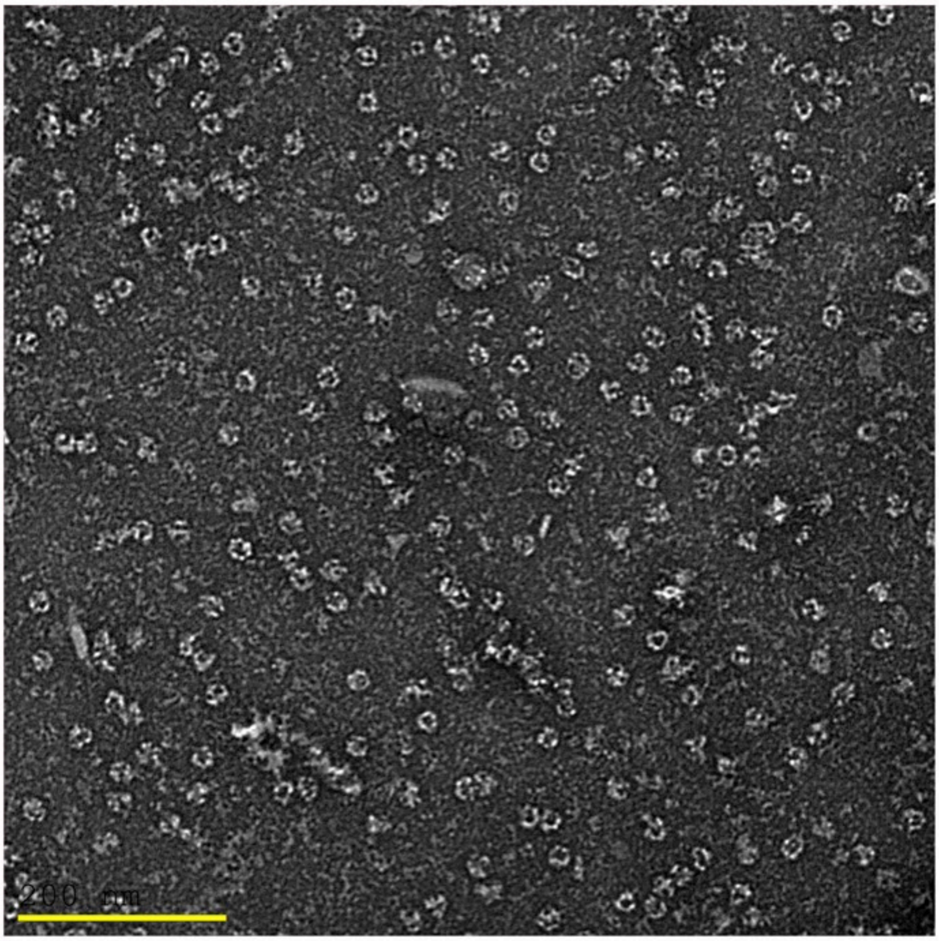

Supplementary Figure 1 | Homogeneous appearance of FAS-I particles

Micrograph of purified FAS-I visualized with negative stain. Scale bar represents 200 nm.

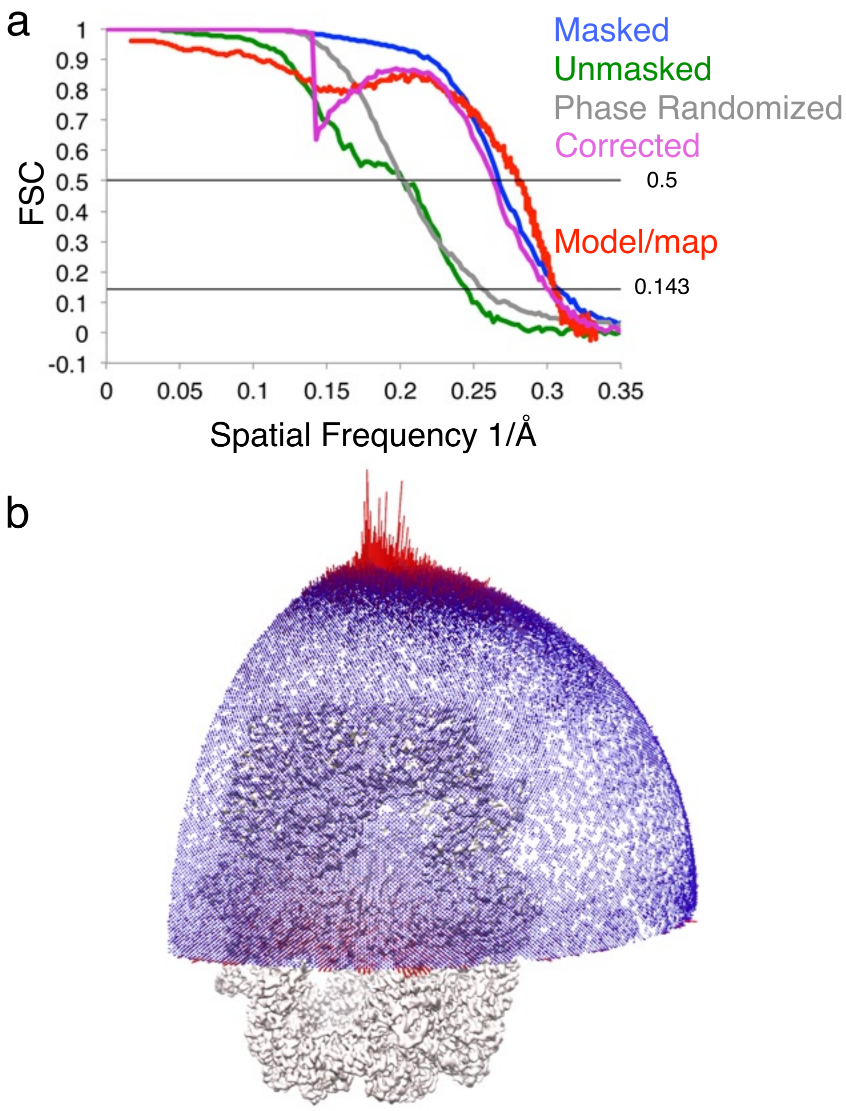

Supplementary Figure 2 | Resolution and completeness of the 3D reconstruction

a, Fourier shell correlations (FSC) between two unfiltered half maps, with and without solvent masking (blue and green, respectively), between two half maps with randomized phases (grey), and masked-corrected correlation (purple). FSC between the model and the map is shown in red. **b**, Angular coverage for the map reconstruction using D3 symmetry. The heights of the spikes are proportional to the orientation frequencies.

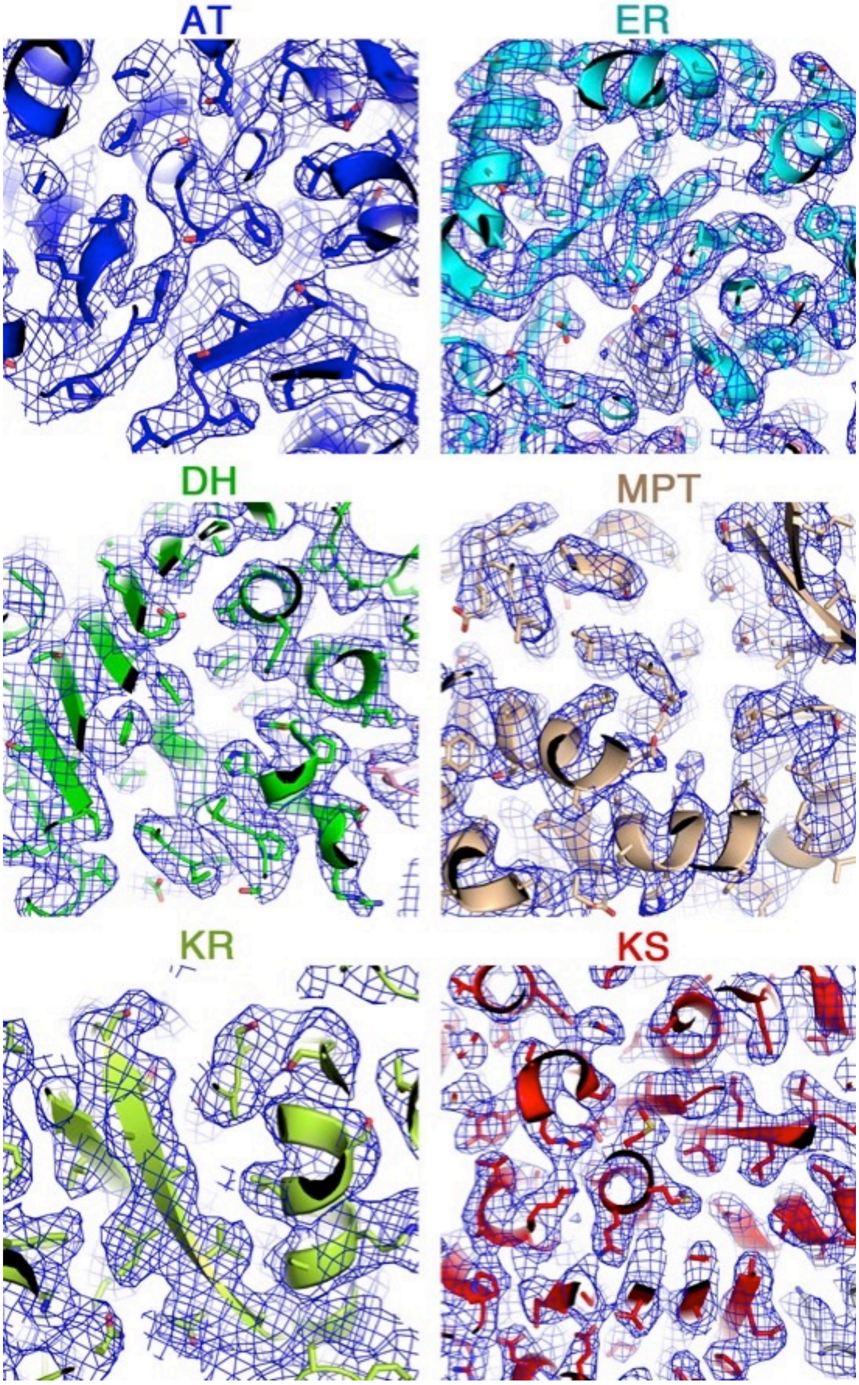

Supplementary Figure 3 | Electron density at core regions of the catalytic domains

Electron density at σ=4 is shown as blue mesh at the six catalytic domains that were modeled in the *Mtb* FAS-I structure. Each catalytic domain is shown using a distinct color: acetyltransferase (AT) in blue, enoyl reductase (ER) in cyan, dehydratase (DH) in green, malonyl transacylase (MPT) in brown, ketoacyl reductase (KR) in light green, and ketoacyl synthase (KS) in red.

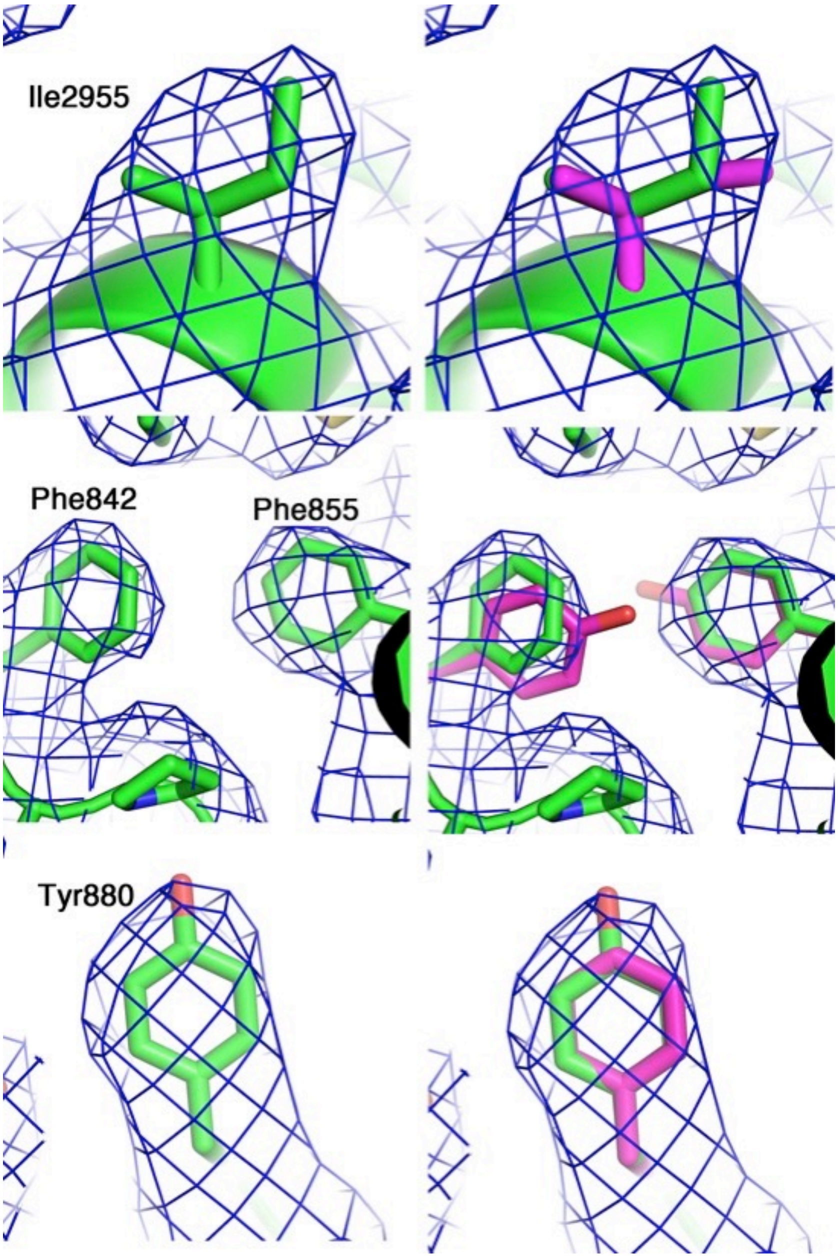

Supplementary Figure 4 | Quality of electron density at core regions allow rotamer assignments as well as to distinguish between amino acids with similar shapes

Electron density at σ=4 is shown as blue mesh. Left column shows modeled amino acids (green sticks) and the right column illustrates erroneously modeled rotamer / residues (magenta). At inner regions, the quality of density is sufficient to determine the correct rotamer of isoleucine residues (top row), as well as to distinguish between phenylalanine and tyrosine residues (middle and bottom rows, respectively).

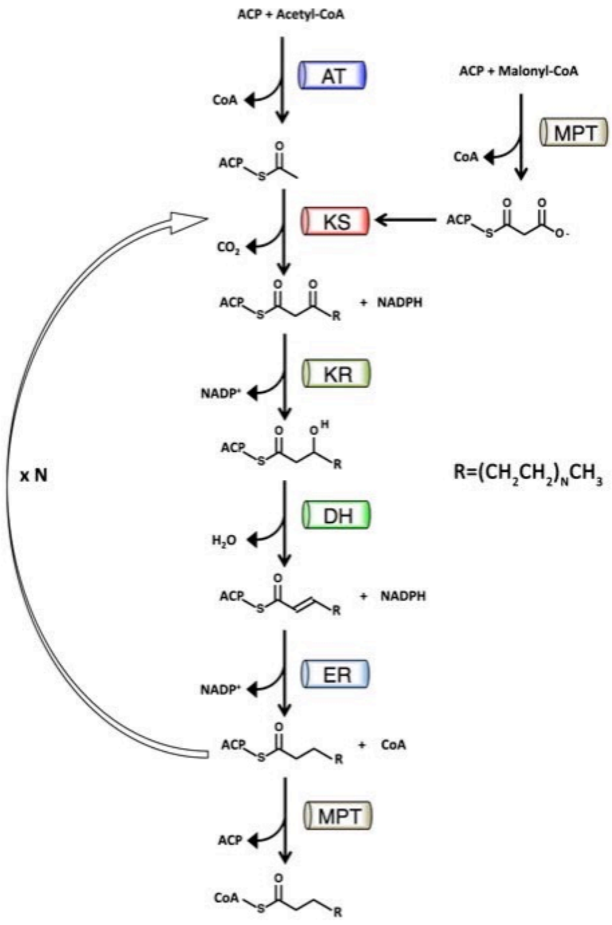

Supplementary Figure 5 | Schematic diagram of the FAS-I catalytic cycle

Transfer of an acetyl group from CoA to ACP by the AT domain initiates fatty acid synthesis. MPT loads a malonyl group on ACP to feed a decarboxylative condensation reaction catalyzed by KS. Subsequently, a sequence of reduction (KR), dehydration (DH), and another reduction step (ER) creates a saturated bond. This sequence of reactions effectively adds to the initial acetyl group two additional carbons. This cycle repeats until the final product is obtained. MPT releases the fatty acid once its reaches the final length, which is up to C_{26} for *Mtb*-FAS-I.

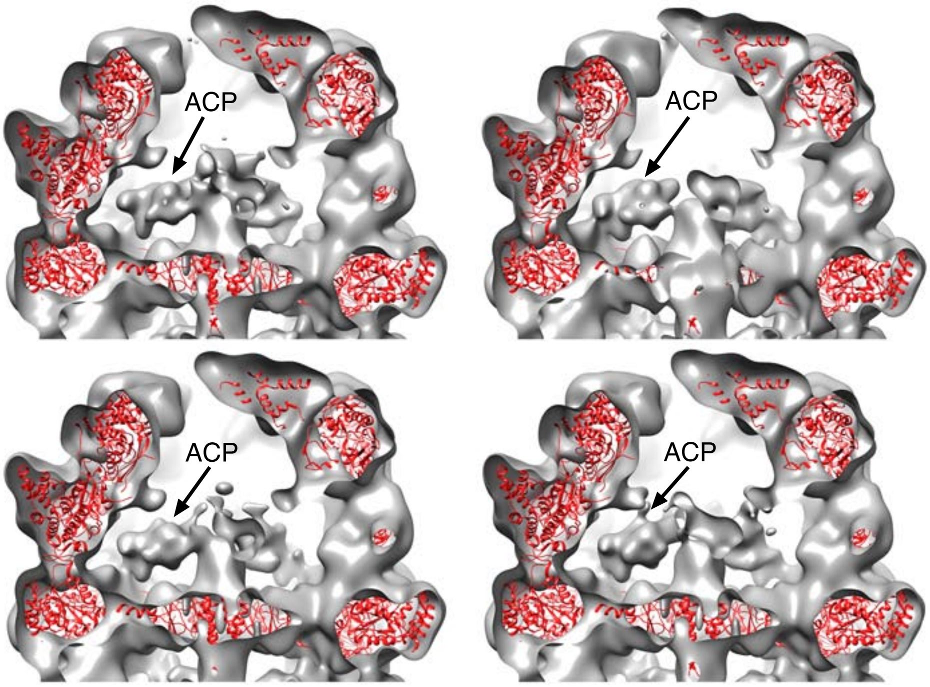

Supplementary Figure 6 | Variations in the location of ACP across different 3D classes

Cross sections through four different 3D classes low-pass filtered to 15 Å (an additional 3D class is shown as part of figure 2). The *Mtb* FAS-I model is shown as red ribbon. In each of the maps the ACP domains adopt different orientations but they generally orient toward the KS domains.

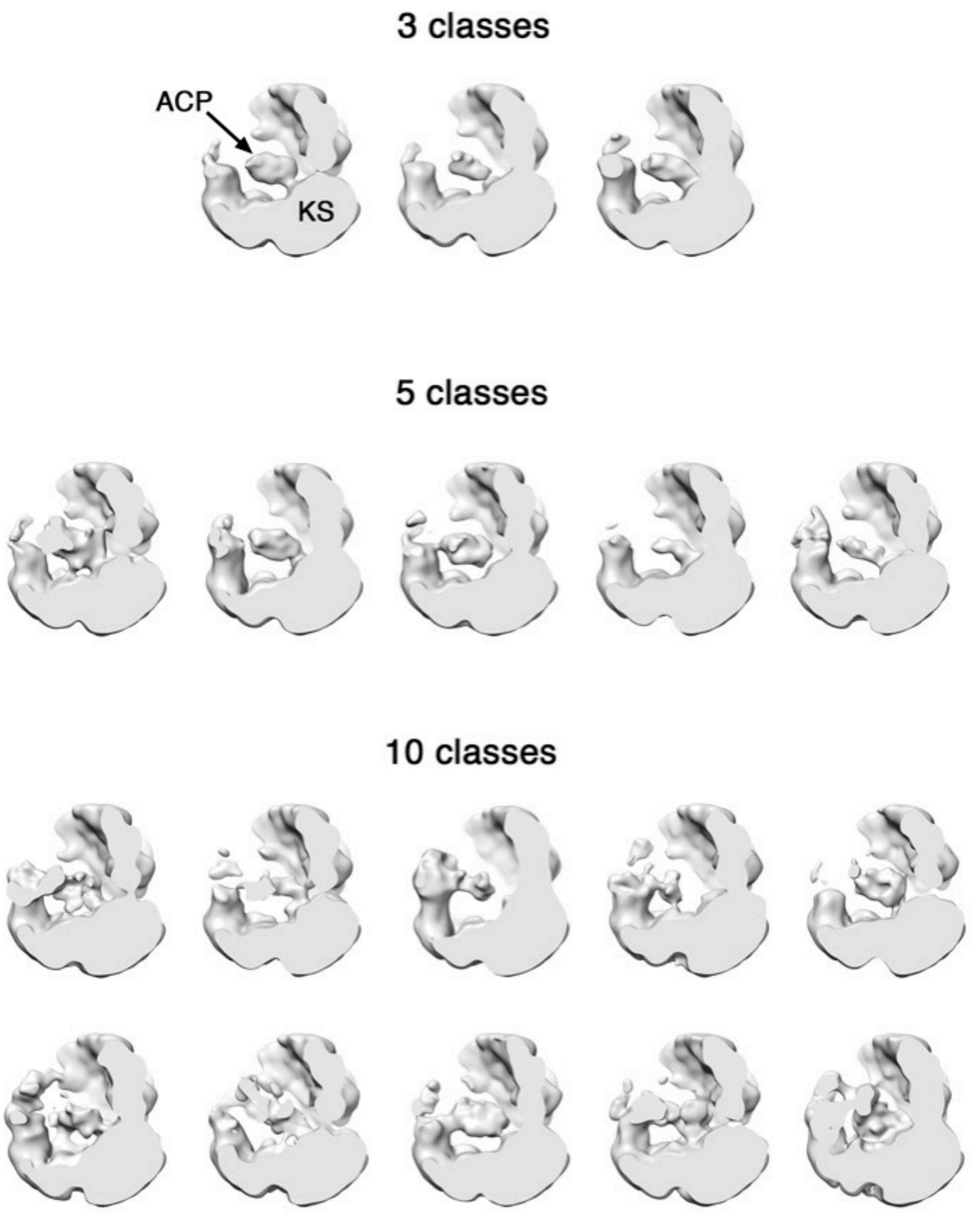

Supplementary Figure 7 | ACP adopts multiple orientations at the vicinity of the KS module

Localized reconstruction[1] analysis of the ACP domain. ACP domain sub-particles were classified into 3, 5, and 10 classes at the top, middle, and bottom panels, respectively. The KS module as well as the ACP domain are indicated in the upper left

reconstruction. The ACP is highly mobile although its location is restricted within the chamber to the vicinity of the KS.

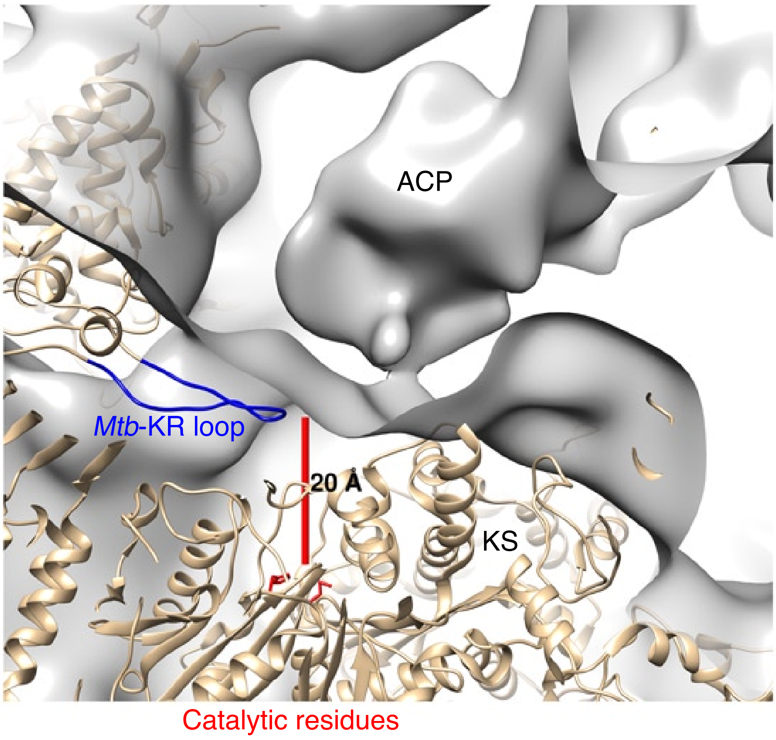

Supplementary Figure 8 | A loop projecting from the KR module forms part of the ACP binding site on KS

A low-pass filtered map from a 3D-class of *Mtb* FAS-I is shown using a grey surface. A gold-colored ribbon shows the KS domain of *Mtb* FAS-I. The catalytic residues are shown using red sticks, and a loop projecting from the KR domain toward the opening of the KS active site is colored blue. This long loop forms part of the ACP docking site, as it inserts between the ACP and the surface of KS. A 20 Å red scale bar is included. A 3D representation of this image is included as Extended Data Movie 9.

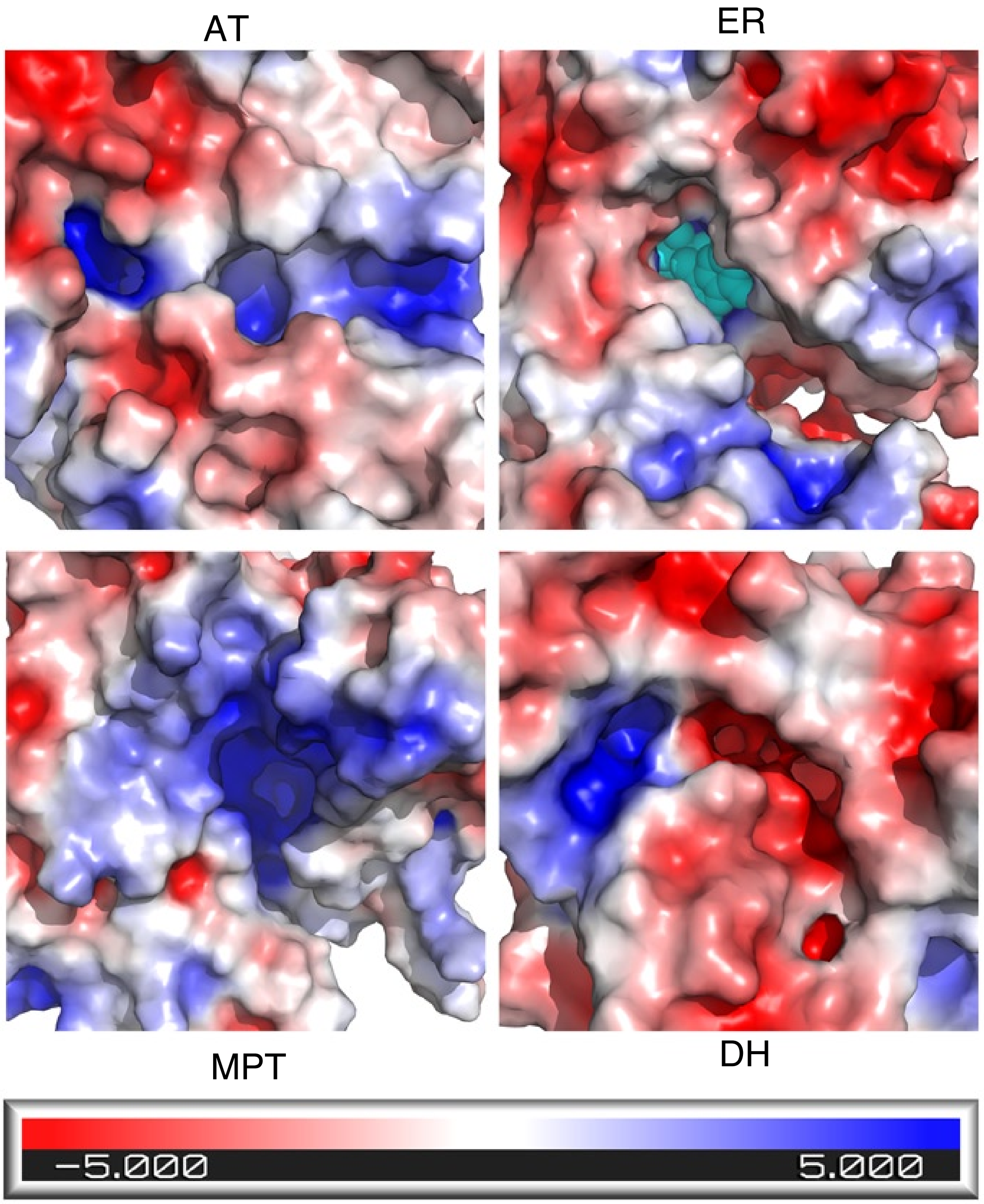

Supplementary Figure 9 | Negatively charged surface electrostatic potentials are a feature of some but not all of the catalytic modules of *Mtb* FASI

Electrostatic potentials at the solvent accessible surfaces mapped on the molecular surfaces of the AT, ER, MPT, and DH modules are shown as indicated. Negatively charged areas are in red (-5.0 kT/e) and positively charged areas are in blue (+5.0 kT/e). Electrostatic potentials were calculated using APBS tools[2]. The catalytic sites of all modules are centered. Except in MPT, negative charge is dominating the vicinity of the active sites of the various modules.

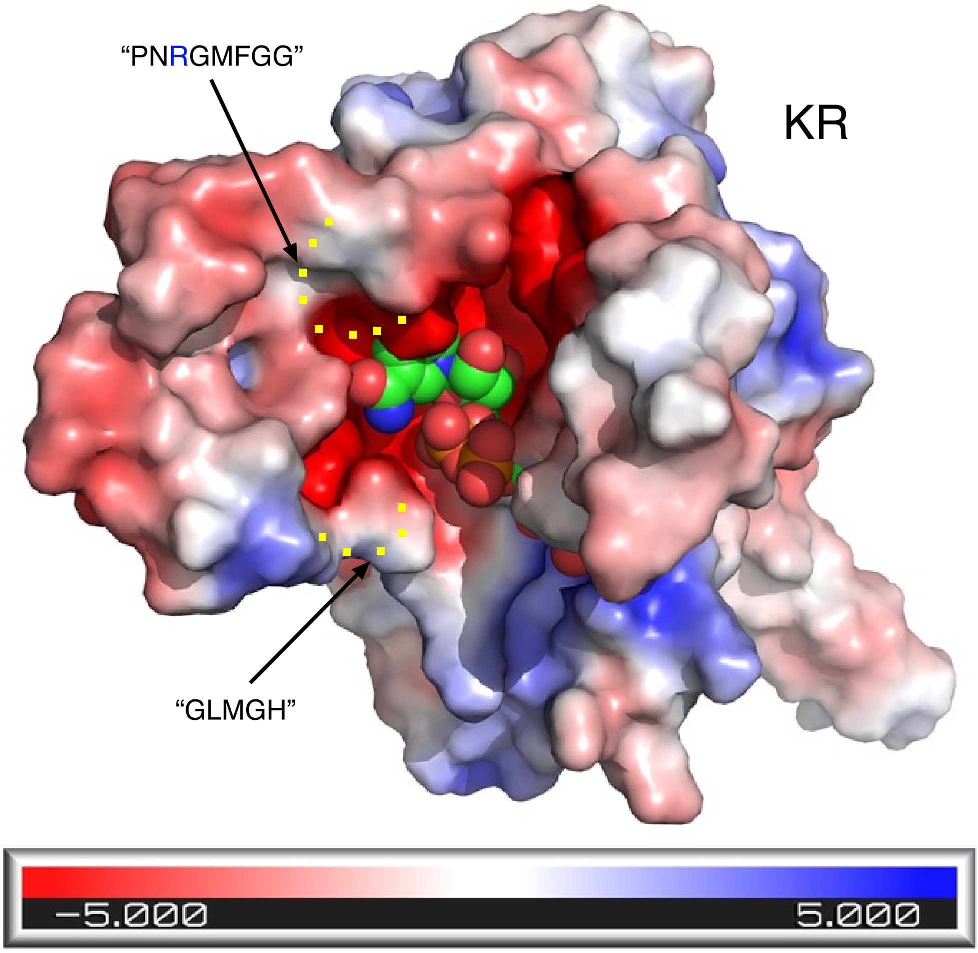

Supplementary Figure 10 | Negatively charged surface of the KR module near its active site

Electrostatic potential at the solvent accessible surface mapped on the molecular surface of the KR module is shown. Negatively charged areas are in red (-5.0 kT/e) and positively charged areas are in blue (+5.0 kT/e). Electrostatic potentials were calculated using APBS tools[2]. The NADPH from the structure of fungal FAS-I (PDB: 4V59) is superimposed on the structure of *Mtb* FAS-I and is shown in green spheres. Two loops at the vicinity of the catalytic cleft had missing density and hence were not modeled. Yellow dots represent the likely path of these two loops. The amino acid sequences of the missing loops are indicated. Except for a single positively charged arginine in one loop, no other charged residues are missing from the model. The overall electrostatic potential near the active cleft seems to be negative.

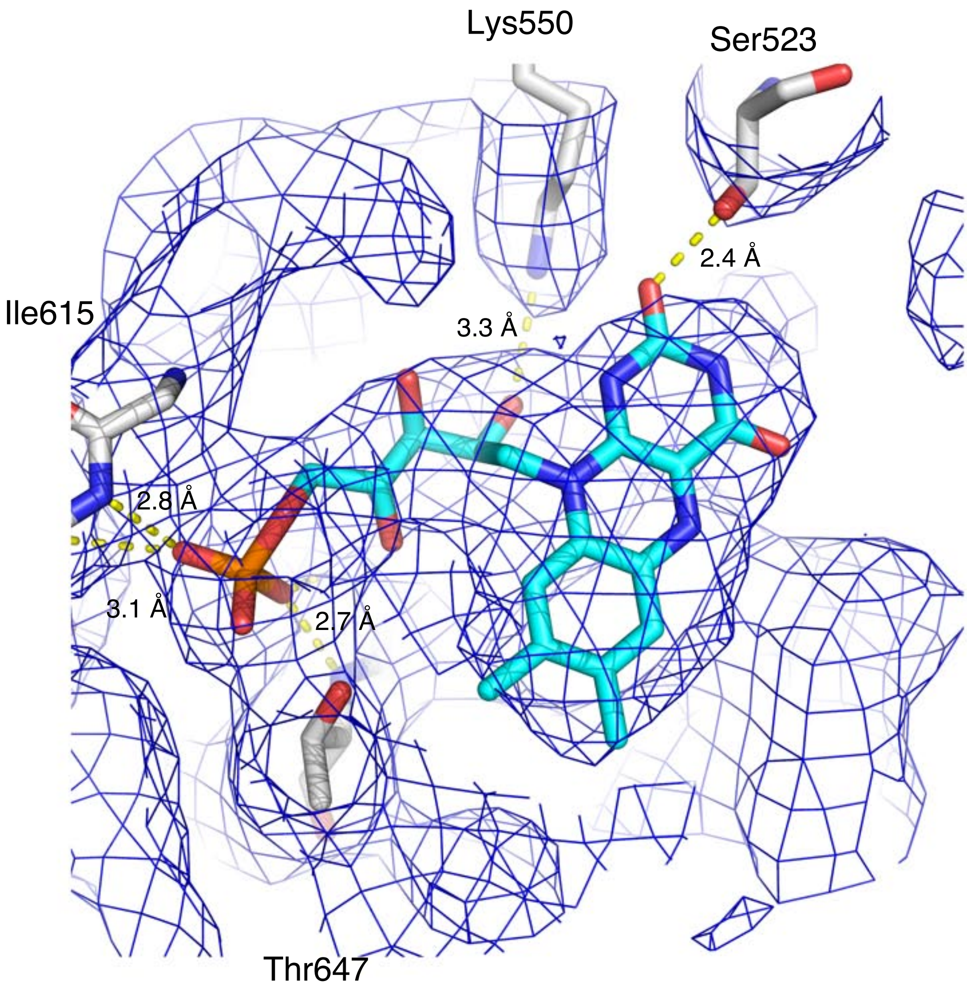

Supplementary Figure 11 | Density for the FMN co factor and surrounding residues

The FMN co factor of *Mtb*-ER is shown as sticks in cyan. Density map at σ=4 is shown as blue mesh. The FMN is stabilized by multiple hydrogen bonds. The phosphate group is coordinated by four backbone amide groups; two at the N terminus of a helix, for which the phosphate group is serving as an N-cap (Thr647 is visible), and two from a nearby loop (Ile615 is visible). Also, FMN makes hydrogen bonds with the side chains of Ser523 and Lys550, which are accurately positioned in the density map.

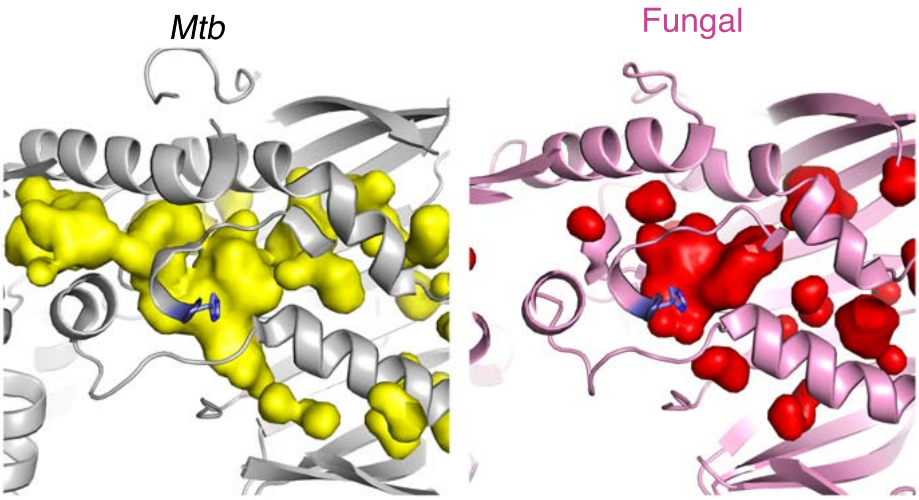

Supplementary Figure 12 | The inner cavity of the *Mtb* DH is larger compared to the fungal counterpart

Ribbon representations of *Mtb* FAS-I (grey) and fungal FAS-I (pink, PDB code: 4v59). The inner cavities of the catalytic sites were delimited using the same threshold and are shown as yellow and red surfaces for *Mtb* FAS-I and fungal FAS-I, respectively. The catalytic histidine residues are shown in blue.

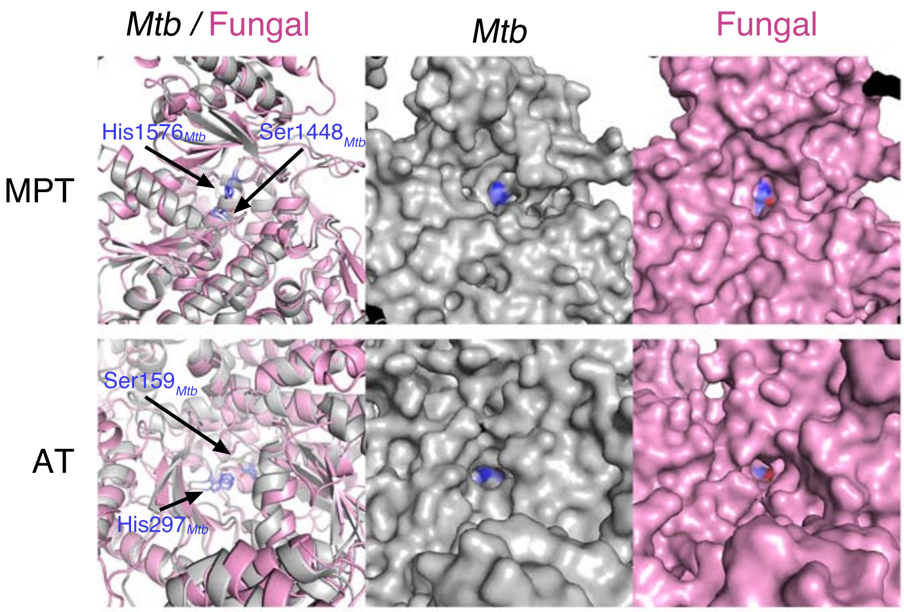

Supplementary Figure 13 | Catalytic clefts of the ACP-loading modules

The two ACP-loading domains are shown using ribbon representations (left column). The polypeptide chains of *Mtb* FAS-I (grey) and of the fungal FAS-I (pink, PDB code: 4V59) are superimposed. The middle and right columns show surface representations of *Mtb* FAS-I and the fungal FAS-I, respectively. The malonyl transacylase domains (MPT, upper row), as well as the acetyltransferase domains (AT, lower row), are shown with the catalytic histidine and serine residues in blue.

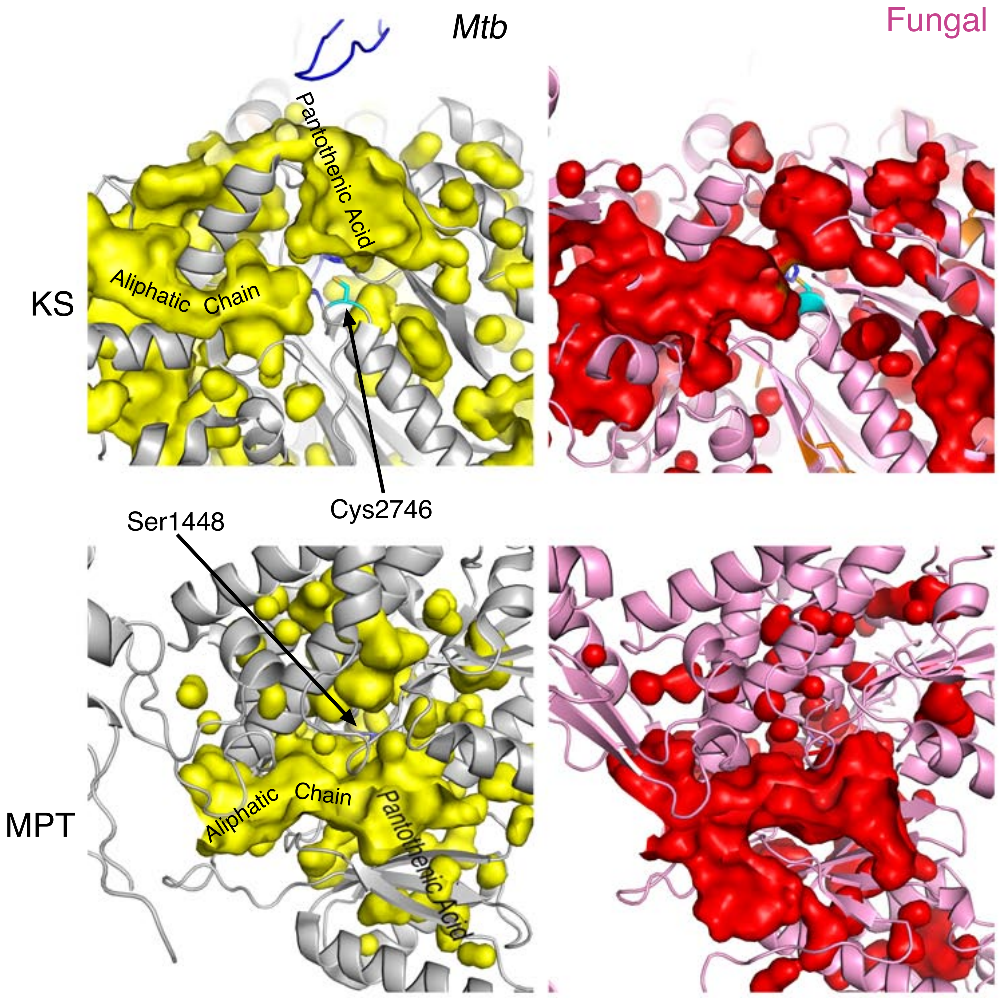

Supplementary Figure 14 | Inner cavities of both KS and MPT can accommodate long aliphatic chains

Surface representations of the inner cavities from the KS modules (upper row) and the MPT modules (lower row) of *Mtb* (left) and of the fungal (PDB: 4V59) FAS-I are shown on top of a ribbon model of the proteins. A suggested space that the growing aliphatic chains of the fatty acid can accommodate is indicated on the *Mtb* derived modules. Cys2746 of the *Mtb*-KS module, which carries the growing fatty acid during the catalytic cycle, is indicated. Although the density in the vicinity of this cysteine is good, the density for this residue is poor, indicating high mobility. In both Mtb and fungal KS modules, the channels that potentially accommodate the aliphatic chains of

the fatty acids are opened to solvent and thus should not restrict the length of these chains. The presumed space of the pantothenic acid arm that connects the ACP to the growing fatty acid is also indicated. For the MPT modules, long clefts that are also open to solvent can be seen in both cases. The catalytic serine residue of *Mtb*-MPT is indicated, as well as the potential regions that could accommodate the pantothenic acid and the aliphatic chain.

Supplementary Table 1 – Model & Quality Parameters

**Refinement**	
Model to Map correlation	0.8282
Model	
Protein atoms	124806
Ligand atoms	186
Average B factors	
Protein	108.3
Ligand	68.0
**Geometry Analysis**	
Ramachandran	
Favored (%)	92.54
Allowed (%)	7.46
Outlier (%)	0.0
Rotamers	
Favored (%)	93.4
Poor (%)	1.03
Root mean square deviations	
Bond length (Å)	0.008
Bond angles (°)	1.04
**MolProbity Analysis**	
Score (percentile)	1.77 (87th)
Clashscore (percentile)	5.48 (92nd)

SUPPLEMENTARY REFERENCES

1 Ilca, S. L. *et al.* Localized reconstruction of subunits from electron cryomicroscopy images of macromolecular complexes. *Nat Commun* **6**, 8843, (2015).

2 Jurrus, E. *et al.* Improvements to the APBS biomolecular solvation software suite. *Protein science : a publication of the Protein Society* **27**, 112-128, (2018).
