# Supplementary material for: Structure of Type-I Mycobacterium tuberculosis fatty acid synthase at 3.3 Å resolution
Source: Nat Commun. 2018 Sep 24;9:3886. doi: 10.1038/s41467-018-06440-6 (PMC6155276; doi:10.1038/s41467-018-06440-6)
Supplement: Supplementary file 3 — Description of Additional Supplementary Files [file 41467_2018_6440_MOESM3_ESM.pdf]

Description of Additional Supplementary Files

File Name: Supplementary Movie 1
Description: Overview of the catalytic pocket of the Mtb – ER module
The ER module of Mtb FAS-I is shown in grey surface representation. The FMN cofactor is shown in cyan-colored sticks, docked at the catalytic cleft. The movie shows a 30° rotational tilt of the structure.

File Name: Supplementary Movie 2
Description: Overview of the catalytic pocket of the Fungal – ER module
The ER module of fungal FAS-I (PDB code: 4V59) is shown in pink surface representation. Both the FMN and NADPH co-factors are shown as sticks in cyan and green, respectively. The movie shows a 30° rotational tilt of the structure.

File Name: Supplementary Movie 3
Description: Overview of the catalytic pocket of the Mtb – KR module
The KR module of Mtb FAS-I is shown in grey surface representation. The NADPH cofactor from the fungal FAS-I (PDB code: 4V59, shown as green spheres) is included to help visualize the catalytic cleft. A loop that partially conceals the catalytic cleft and changes conformation in respect to the fungal KR (residues 2237-2248) is highlighted in black. The movie shows a 30° rotational tilt of the structure.

File Name: Supplementary Movie 4
Description: Overview of the catalytic pocket of the Fungal – KR module
The KR module of fungal FAS-I (PDB code: 4V59) is shown in grey surface representation. The NADPH co-factor is shown as green spheres. A loop that partially conceals the catalytic cleft and changes conformation in respect to the Mtb KR (residues 750-761) is highlighted in hot pink. The movie shows a 30° rotational tilt of the structure.

File Name: Supplementary Movie 5
Description: Overview of the catalytic pocket of the Mtb – DH module
The DH module of Mtb FAS-I is shown in grey surface representation. The catalytic histidine is shown in blue inside the cleft. The movie shows a 30° rotational tilt of the structure.

File Name: Supplementary Movie 6
Description: Overview of the catalytic pocket of the Fungal – DH module
The DH module of fungal FAS-I (PDB code: 4V59) is shown in pink surface representation. The catalytic histidine is shown in blue inside the cleft. The movie shows a 30° rotational tilt of the structure.

File Name: Supplementary Movie 7
Description: Overview of the catalytic pocket of the Mtb – KS module
The KS module of Mtb FAS-I is shown in grey surface representation. The catalytic histidine and lysine residues are shown in blue inside the cleft. A long loop projected from the KR module and forms part of the ACP interaction surface is colored black. The movie shows a 30° rotational tilt of the structure.

File Name: Supplementary Movie 8
Description: Overview of the catalytic pocket of the Fungal – KS module
The KS module of fungal FAS-I is shown in pink surface representation. The catalytic histidine and lysine residues are shown in blue inside the cleft. The short equivalent loop to the Mtb-KR loop is colored hot pink. The movie shows a 30° rotational tilt of the structure.

File Name: Supplementary Movie 9
Description: Overview of the KR projecting loop near the KS active site
The Mtb-KR loop is shown in blue. The histidine and lysine catalytic residues of the KS module are shown in red and density for the ACP domain is shown in purple. A 360° rotation is shown to illustrate how the loop will affect the binding of the ACP domain.
